# Identifying instruments for measuring agitation and other non-cognitive symptoms in people with advanced dementia in residential settings: a scoping review protocol

**DOI:** 10.1136/bmjopen-2024-096540

**Published:** 2025-08-12

**Authors:** Mary Faherty, Lauren O' Mahony, Nicola Cornally, Noeleen Brady, Caroline Dalton O’ Connor, Siobhan Fox, Irene Hartigan, Brenda van den Broek, Jenny T van der Steen, Suzanne Timmons

**Affiliations:** 1 Centre for Gerontology and Rehabilitation, University College Cork, Cork, Ireland; 2 Catherine McAuley School of Nursing and Midwifery, University College Cork, Cork, Ireland; 3 Radboudumc Alzheimer Centre and Department of Primary and Community Care, Radboud University Medical Centre, Nijmegen, The Netherlands; 4 Department of Public Health and Primary Care, Leiden University Medical Centre, Leiden, The Netherlands; 5 Cicely Saunders Institute, King’s College London, London, UK

**Keywords:** Dementia, Behavior, Nursing Homes, Prevalence, Psychometrics, Systematic Review

## Abstract

**Abstract:**

**Introduction:**

Various instruments exist for assessing agitation and broader non-cognitive symptoms in dementia (NCSD). However, the feasibility and practicality of using these instruments in residential settings with people with advanced dementia have not been evaluated. The aim of our review is to identify the available evidence regarding tools for measuring (1) Agitation and (2) NCSD in people with advanced dementia in residential settings, in terms of use (feasibility and psychometric properties) in this population.

**Methods and analysis:**

Literature searches will be carried out in Medline, Embase, CINAHL, PsycInfo, Scopus, Cochrane Database of Systematic Reviews and Cochrane Central Register of Controlled Trials. Grey literature databases and relevant websites will also be explored for guidance documents, task reports, etc. A three-stage screening process will be adopted and will include pilot testing of source selectors. Two reviewers will independently perform title and abstract screening, then full text screening, against the defined eligibility criteria. This scoping review protocol was registered with Open Science Framework (https://osf.io/p7g86).

**Ethics and dissemination:**

Due to the nature of the scoping review, ethical approval is not required. Results will be disseminated in a peer-reviewed journal and at international conferences.

**STRENGTHS AND LIMITATIONS OF THIS STUDY**

- The scoping review will adhere to best practice, using the Joanna Briggs Institute methodology for scoping reviews and the Preferred Reporting Items for Systematic reviews and Meta-Analyses extension for Scoping Reviews Checklist.
- By informing practitioners and researchers on the current state of play, this study will provide a broad map for future research in this area.
- This study focuses on a very select population (ie, people with advanced dementia in residential care).
- If the available evidence is limited or of moderate quality, it may limit the value and uptake of the study findings.

## Introduction

 Neuropsychiatric symptoms (NPS) of dementia, also known as behavioural and psychological symptoms of dementia, and more recently as non-cognitive symptoms of dementia (NCSD), can often be distressing for the person experiencing the symptoms and for those providing care.^1^ Agitation is cited as one of the most common NPS of dementia^2^,^4^ and manifests as excessive motor activity, verbal aggression or physical aggression.^5^ As dementia progresses to an advanced stage, agitation becomes more prevalent and challenging to manage,^3^ though it declines in the last week of life.^6^ Agitation can negatively impact the quality of life of both residents and carers in nursing homes.^7 8^ A systematic review reported the worldwide *prevalence* of agitation in people with dementia to range from 5%–88% across all studies and care settings, and from 24%–88% in nursing homes and geriatric facilities in Europe.^9^ Prospective cohort studies of nursing home populations, over 16–24 months, found agitation cumulative *incidence* rates of 24%–42%.^4 10^

While there are many tools to measure agitation, the Cohen-Mansfield Agitation Inventory (CMAI), the Pittsburgh Agitation Scale (PAS) and the Richmond Agitation Sedation Scale (RASS) are among the most popular. The CMAI focuses specifically on agitation, measuring 29 behaviours.^11 12^ It has been used in several randomised controlled trials to measure agitation as an outcome in people with dementia in care homes.^13 14^ The PAS categorises agitation into four groups: aberrant vocalisation, motor agitation, aggressiveness and resisting care.^15^ The RASS originated in critical care settings, where it is widely used, and is sometimes used for delirium detection in people with dementia.^16^ Other tools include the agitated behaviour in dementia scale, behavioural activity rating scale and the minimum data set agitated and reactive behaviour scale.^17^,^19^

Apart from agitation, other common NCSDs are depression, anxiety, apathy and psychosis, which often manifest as behaviour change.^20^ Various scales and tools have been developed to measure NCSD. The most common is the Neuropsychiatric Inventory (NPI), a broad-spectrum scale that assesses twelve NPS, including agitation.^21^ It is often used in clinical trials to assess NPS as an outcome.^22^ A specific version was developed for use in nursing homes, the NPI-NH, which is administered by professional carers.^23^

Several other scales exist for assessing NCSD, which vary in terms of target settings (hospital, community or nursing home), focus, complexity, assessment duration, instrument administrator, reliability and validity. Many focus on behaviours, such as the Behavioral Pathology in Alzheimer's Disease Rating Scale (BEHAVE-AD), the Neurobehavioral Rating Scale (NBRS), the nursing home behaviour problem scale, the behaviour rating scale for dementia and the revised memory and behaviour problems checklist.^2224^,^28^

A 2024 systematic review on diagnostic tools for measuring *agitation and aggression* in dementia identified six studies that assessed diagnostic accuracy for detecting agitation. The review found the Spanish NPI, NBRS and PAS had the highest sensitivity and thus diagnostic accuracy for detecting agitation in people with dementia.^29^ While highly relevant to this scoping review, this systematic review considered all settings, including hospital and community settings, and dementia severity was only reported in half the studies, with little selective data from people with ***advanced dementia*** in residential settings available. Due to profound motor and verbal impairment, it cannot be assumed that tools will perform the same in people with advanced dementia, who may not be physically able to express a delusion, make verbal sexual advances, ask repetitive questions, aimlessly wander, hide things, complain or be negative, etc, (CMAI items used here as examples). In addition, we will consider tools for assessing NCSD other than agitation and aggression and will consider their feasibility and psychometric parameters, thus broadening the scope beyond that systematic review.

This scoping review aims to identify the tools currently available for measuring (1) Agitation and (2) NCSD in people with advanced dementia in residential settings, and the use and usefulness in this population. The latter includes feasibility and ease of use, diagnostic accuracy, sensitivity to change and other psychometric parameters. The tools are relevant to both research and practice, with research instruments designed to measure specific variables or concepts in a detailed and systematic way, while clinical screening tools focus on identifying people at risk of a condition who may need further evaluation. The use of appropriate, valid and reliable tools is key to ensuring people with advanced dementia in nursing homes can be accurately screened and can thus avail of appropriate pharmacological and non-pharmacological treatments that lead to improved care outcomes. Equally accurate and reliable measurement of outcomes is essential for trials to demonstrate intervention effectiveness.

A further research focus for this review is to examine the incidence and prevalence of agitation and other NCSD in people with advanced dementia in residential care. This informs the sample size needed to detect an improvement in such symptoms with a therapeutic intervention in this population. We hypothesise that agitation and other NCSD may be less commonly expressed in advanced dementia (for reasons mentioned above), such that they require an appropriately powered sample to detect change.

## Study objective

The scoping review aims to identify assessment tools for agitation and for broader NCSD that are appropriate for use with people with advanced dementia in residential care settings.

The specific research questions are:

1. What tools/instruments have been used for assessing (1) Agitation and (2) NCSD in people with advanced dementia in residential care settings?
2. Are these tools feasible to use in this population?
3. Are these tools accurate and sensitive to change in this population?
4. What is the incidence and prevalence of agitation and NCSD using these tools?

## Methods

The study will use the Joanna Briggs Institute methodology for scoping reviews.^30^ Reporting will comply with the 20 essential reporting items listed in the Preferred Reporting Items for Systematic reviews and Meta-Analyses extension for Scoping Reviews (PRISMA-ScR) checklist.^31^

The expected study duration is October to December 2024.

### Search strategy

A three-step search strategy will be used. First, an initial limited search of two databases (Medline and CINAHL) will be carried out to facilitate an analysis of text words contained in the title and abstract of retrieved papers, as well as MeSH terms used to describe the articles, to improve the search terms. A second search using all identified keywords and index terms will then be undertaken across all selected databases. Thirdly, the reference lists of all included articles will be searched for additional sources. If relevant, the researchers will contact authors of primary sources or reviews for further information.

The following electronic databases will be searched: Medline, Embase, CINAHL, PsycInfo, Scopus, Cochrane Database of Systematic Reviews and Cochrane Central Register of Controlled Trials. Grey literature sources will include Google, Google Scholar and Turning Research Into Practice (TRIP).

A complete search strategy is included as a supplement to the protocol. Search terms were derived from the research question, with initial search strings developed by the first reviewer and verified against the 2015 peer review of electronic search strategies checklist.^32^

### Source of evidence selection

Article selection will be based on the pre-specified inclusion criteria in this protocol. Rayyan and Zotero will be used to manage the results. Pilot testing will be carried out to ensure the screening process is conducted accurately and consistently. Two reviewers will screen the title and abstract. They will screen a random sample of 25 titles/abstracts, then meet to discuss discrepancies between their decisions and modify the eligibility criteria or definitions if necessary. When the reviewers agree on the screening process, formal title/abstract screening will commence. For pilot testing of full-text screening, another random sample of 25 articles will be screened by two reviewers. Once again, these reviewers will meet to discuss discrepancies, and when both reviewers are aligned, an independent full-text examination will begin. Any disagreements at any screening stage will be resolved by consensus meetings, or if necessary, by the decision of a third (senior) reviewer.

### Data extraction/data charting

A draft charting table was developed (online supplemental file 1) based on the PRISMA-ScR checklist^31^ and the scoping review guidance in Peters *et al*.^30^ This will be further refined and updated at the review stage if required. Extracted information will include author(s), title, year of publication, country of origin, aims/purpose, population and sample size, methodology, intervention type (if relevant), instrument characteristics and details, outcomes and key findings relevant to the scoping review research questions, including the percentage of the population experiencing NCSD and/or agitation, pre-data and post-data in control and intervention groups (where relevant) for sensitivity to change. Extracted data will be presented in tables.

### Inclusion criteria

The population-concept-context framework^30^ was used as the basis for defining the inclusion criteria summarised in table 1.

**Table 1:** Inclusion criteria

Population	Adults with advanced dementia, defined as physical and cognitive disability due to a neurodegenerative disease and corresponding to a score of 6 or 7 using either the functional assessment staging tool scale or the global deterioration scale, or a mini-mental state exam score of <10, or an equivalent marker of advanced dementia as judged by the review team
Concept of interest	Sources which use tools/instruments to measure agitation and/or NCSD through directly observed, or staff-reported outcome measures; focus on validity, sensitivity to change, ease and completeness of data collection. Agitation is defined as *“(1) Occurring in patients with a cognitive impairment or dementia syndrome, (2) Exhibiting behaviour consistent with emotional distress, (3) Manifesting excessive motor activity, verbal aggression or physical aggression and (4) Evidencing behaviours that cause excess disability and are not solely attributable to another disorder (psychiatric, medical or substance-related)”*. Cummings *et al*, 2015, p.7 NCSD includes agitation, aberrant motor behaviour, anxiety, elation, irritability, depression, apathy, disinhibition, delusions, hallucinations and sleep or appetite changes
Context	Nursing homes, residential care facilities and long-term care settings providing care to older or general adult populations. These include specific dementia units but exclude specialised units for people with intellectual disability or acquired brain injury
Types of evidence sources	Randomised controlled trials (RCTs), non-RCTs, quasi-experimental before and after studies, prospective cohort studies, retrospective cohort studies, observational studies, case-control studies
Source characteristics	Published since 1 January 2000; articles in any language

NCSD, non-cognitive symptoms of dementia.

### Patient and public involvement

Patients and/or the public were not involved in the design, conduct, reporting or dissemination plans of this research.

## Ethics and dissemination

Research ethics approval and consent are not required for this scoping review. The findings will be made available to health professionals, decision makers and the public via publication in relevant journals and dissemination at regional, national and international conferences.

## Supplementary material

10.1136/bmjopen-2024-096540 online supplemental file 1
