# Supplementary material for: Identifying instruments for measuring agitation and other non-cognitive symptoms in people with advanced dementia in residential settings: a scoping review protocol
Source: BMJ Open. 2025 Aug 12;15(8):e096540. doi: 10.1136/bmjopen-2024-096540 (PMC12352213; doi:10.1136/bmjopen-2024-096540)
Supplement: online supplemental file 1 [file bmjopen-15-8-s001.docx]

# **Identifying instruments for measuring agitation and other non-cognitive symptoms in people with advanced dementia in residential settings: a scoping review protocol**

**Supplementary File 1**

**Table 1: Medline Search Term**

|  | KEY CONCEPTS | | | |
| --- | --- | --- | --- | --- |
| MeSH / Subject Headings | Dementia | Residential Facilities | Behavioral Symptoms | - |
| Keywords | ‘‘advanced dementia’’ OR “late* stage dementia” OR “severe dementia” OR “end* stage dementia” OR “advanced Alzheimer*” OR “late* stage Alzheimer*” OR “severe Alzheimer*” OR “end* stage Alzheimer*” OR “lewy bod*” OR “frontotemporal” OR “vascular dementia” | “long-term care setting*” OR “nursing home*” OR “residential care setting*” OR “Residential facilit*” OR “institution* care” OR “nursing care” OR “nursing facilit*” OR “continuous care” OR “elderly care facilit*” | “behav* rating scale” OR “behav*” OR “non-cognitive symptom*” OR “non cognitive symptom*” OR “Delusion*” OR “Hallucination*” OR “Agitat*” OR “Aggression” OR “Depression” OR “Dysphoria” OR “Anxiety” OR “Elation” OR “Euphoria” OR “Apathy” OR “Indifference” OR “Disinhibition” OR “Irritability” OR “Lability” OR “Aberrant Motor Behavio*” OR “sleep” OR “appetite” | “tool” OR “instrument” OR “assessment” OR “screening” OR “inventory” |

**Table 2: Embase Search Term**

| EMBASE | Dementia | Residential setting | NPS /NCSD / BPSD or Agitation | Tool |
| --- | --- | --- | --- | --- |
| Emtree Headings | ‘dementia’ | ‘residential home’ | - | - |
| Keywords | ‘advanced dementia’ OR ‘late* stage dementia’ OR ‘severe dementia’ OR ‘end* stage dementia’ OR ‘advanced Alzheimer*’ OR ‘late* stage Alzheimer*’ OR ‘severe Alzheimer*’ OR ‘end* stage Alzheimer*’ OR ‘lewy bod*’ OR ‘frontotemporal’ OR ‘vascular dementia’ | ‘long-term care setting*’ OR ‘nursing home*’ OR ‘residential care setting*’ OR ‘Residential facilit*’ OR ‘institution* care’ OR ‘nursing care’ OR ‘nursing facilit*’ OR ‘continuous care’ OR ‘elderly care facilit*’ | ‘behav* rating scale” OR “behav*’ OR ‘non-cognitive symptom*’ OR ‘non cognitive symptom*’ OR ‘Delusion*’ OR ‘Hallucination*’ OR ‘Agitat*’ OR ‘Aggression’ OR ‘Depression’ OR ‘Dysphoria’ OR ‘Anxiety’ OR ‘Elation’ OR ‘Euphoria’ OR ‘Apathy’ OR ‘Indifference’ OR ‘Disinhibition’ OR ‘Irritability’ OR ‘Lability’ OR ‘Aberrant Motor Behavio*’ OR ‘sleep’ OR ‘appetite’ | ‘tool’ OR ‘instrument’ OR ‘assessment’ OR ‘screening’ OR ‘inventory’ |

**Table 3: CINAHL Search Term**

| CINAHL | Dementia | Residential | NPS or Agitation | Tool |
| --- | --- | --- | --- | --- |
| MeSH / Subject Headings | Dementia  Frontotemporal Dementia  Dementia, Vascular  Dementia Patients | Residential Care  Nursing Homes | Subject headings:  Major concept:  Behavior Rating Scales  Agitation | - |
| Keywords | ‘‘advanced dementia’’ OR “late* stage dementia” OR “severe dementia” OR “end* stage dementia” OR “advanced Alzheimer*” OR “late* stage Alzheimer*” OR “severe Alzheimer*” OR “end* stage Alzheimer*” OR “lewy bod*” OR “frontotemporal” OR “vascular dementia” | “long-term care setting*” OR “nursing home*” OR “residential care setting*” OR “Residential facilit*” OR “institution* care” OR “nursing care” OR “nursing facilit*” OR “continuous care” OR “elderly care facilit*” | “behav* rating scale” OR “behav*” OR “non-cognitive symptom*” OR “non cognitive symptom*” OR “Delusion*” OR “Hallucination*” OR “Agitat*” OR “Aggression” OR “Depression” OR “Dysphoria” OR “Anxiety” OR “Elation” OR “Euphoria” OR “Apathy” OR “Indifference” OR “Disinhibition” OR “Irritability” OR “Lability” OR “Aberrant Motor Behavio*” OR “sleep” OR “appetite” | “tool” OR “instrument” OR “assessment” OR “screening” OR “inventory” |

**Table 4: PsycInfo Search Term**

| PSYCINFO | Dementia | Residential setting | NPS /NCSD / BPSD or Agitation | Tool |
| --- | --- | --- | --- | --- |
| APA Thesaurus Subject Headings | Dementia | Nursing Homes or Nursing Home Residents | Psychological Assessment | - |
| Keywords | ‘advanced dementia’ OR ‘late* stage dementia’ OR ‘severe dementia’ OR ‘end* stage dementia’ OR ‘advanced Alzheimer*’ OR ‘late* stage Alzheimer*’ OR ‘severe Alzheimer*’ OR ‘end* stage Alzheimer*’ OR ‘lewy bod*’ OR ‘frontotemporal’ OR ‘vascular dementia’ | ‘long-term care setting*’ OR ‘nursing home*’ OR ‘residential care setting*’ OR ‘Residential facilit*’ OR ‘institution* care’ OR ‘nursing care’ OR ‘nursing facilit*’ OR ‘continuous care’ OR ‘elderly care facilit*’ | ‘behav* rating scale” OR “behav*’ OR ‘non-cognitive symptom*’ OR ‘non cognitive symptom*’ OR ‘Delusion*’ OR ‘Hallucination*’ OR ‘Agitat*’ OR ‘Aggression’ OR ‘Depression’ OR ‘Dysphoria’ OR ‘Anxiety’ OR ‘Elation’ OR ‘Euphoria’ OR ‘Apathy’ OR ‘Indifference’ OR ‘Disinhibition’ OR ‘Irritability’ OR ‘Lability’ OR ‘Aberrant Motor Behavio*’ OR ‘sleep’ OR ‘appetite’ | ‘tool’ OR ‘instrument’ OR ‘assessment’ OR ‘screening’ OR ‘inventory’ |

**Table 5: Scopus Search Term**

| SCOPUS | Dementia | Residential setting | NPS /NCSD / BPSD or Agitation | Tool |
| --- | --- | --- | --- | --- |
| MeSH / Subject Headings | - | - | - | - |
| Keywords | ‘‘advanced dementia’’ OR “late* stage dementia” OR “severe dementia” OR “end* stage dementia” OR “advanced Alzheimer*” OR “late* stage Alzheimer*” OR “severe Alzheimer*” OR “end* stage Alzheimer*” OR “lewy bod*” OR “frontotemporal” OR “vascular dementia” | “long-term care setting*” OR “nursing home*” OR “residential care setting*” OR “Residential facilit*” OR “institution* care” OR “nursing care” OR “nursing facilit*” OR “continuous care” OR “elderly care facilit*” | “behav* rating scale” OR “behav*” OR “non-cognitive symptom*” OR “non cognitive symptom*” OR “Delusion*” OR “Hallucination*” OR “Agitat*” OR “Aggression” OR “Depression” OR “Dysphoria” OR “Anxiety” OR “Elation” OR “Euphoria” OR “Apathy” OR “Indifference” OR “Disinhibition” OR “Irritability” OR “Lability” OR “Aberrant Motor Behavio*” OR “sleep” OR “appetite” | “tool” OR “instrument” OR “assessment” OR “screening” OR “inventory” |

**Table 6: Cochrane Database of Systematic Reviews (CDSR) and Cochrane Central Register of Controlled Trials (CENTRAL)**

|  | Dementia | Residential setting | NPS /NCSD / BPSD or Agitation | Tool |
| --- | --- | --- | --- | --- |
| MeSH / Subject Headings | Dementia | Residential Facilities | Behavioral Symptoms | - |
| Keywords | ‘advanced dementia’ OR ‘late* stage dementia’ OR ‘severe dementia’ OR ‘end* stage dementia’ OR ‘advanced Alzheimer*’ OR ‘late* stage Alzheimer*’ OR ‘severe Alzheimer*’ OR ‘end* stage Alzheimer*’ OR ‘lewy bod*’ OR ‘frontotemporal’ OR ‘vascular dementia’ | ‘long-term care setting*’ OR ‘nursing home*’ OR ‘residential care setting*’ OR ‘Residential facilit*’ OR ‘institution* care’ OR ‘nursing care’ OR ‘nursing facilit*’ OR ‘continuous care’ OR ‘elderly care facilit*’ | “behav* rating scale” OR “behav*” OR “non-cognitive symptom*” OR “non cognitive symptom*” OR “Delusion*” OR “Hallucination*” OR “Agitat*” OR “Aggression” OR “Depression” OR “Dysphoria” OR “Anxiety” OR “Elation” OR “Euphoria” OR “Apathy” OR “Indifference” OR “Disinhibition” OR “Irritability” OR “Lability” OR “Aberrant Motor Behavio*” OR “sleep” OR “appetite” | “tool” OR “instrument” OR “assessment” OR “screening” OR “inventory” |

**Table 7: Grey Literature - TRIP Search Term & Google Scholar Search Term**

| Search Term:  (‘‘advanced dementia’’ OR “late* stage dementia” OR “severe dementia” OR “end* stage dementia” OR “advanced Alzheimer*” OR “late* stage Alzheimer*” OR “severe Alzheimer*” OR “end* stage Alzheimer*” OR “lewy bod*” OR “frontotemporal” OR “vascular dementia”) AND (“long-term care setting*” OR “nursing home*” OR “residential care setting*” OR “Residential facilit*” OR “institution* care” OR “nursing care” OR “nursing facilit*” OR “continuous care” OR “elderly care facilit*”) AND (“behav* rating scale” OR “behav*” OR “non-cognitive symptom*” OR “non cognitive symptom*” OR “Delusion*” OR “Hallucination*” OR “Agitat*” OR “Aggression” OR “Depression” OR “Dysphoria” OR “Anxiety” OR “Elation” OR “Euphoria” OR “Apathy” OR “Indifference” OR “Disinhibition” OR “Irritability” OR “Lability” OR “Aberrant Motor Behavio*” OR “sleep” OR “appetite”) AND (“tool” OR “instrument” OR “assessment” OR “screening” OR “inventory”) |
| --- |

**Table 8: Grey Literature - Google Search Term**

| Search Term:  (‘‘advanced dementia’’ OR “severe dementia” OR “advanced Alzheimer” OR “severe Alzheimer”) AND (“long-term care” OR “nursing home” OR “residential”) AND (“behaviour” OR “behavior” OR “non-cognitive symptom” OR “non cognitive symptom” OR “Agitation”) AND (“tool” OR “instrument” OR “assessment”) filetype:pdf |
| --- |
| Note:  Google limits search queries to 32 words.  Google search does not support * for word truncation |

**Table 9: Draft Charting Table**

| Author(s), Title, Year, Study Location | Aims / purpose of study | Population and sample size | Methodology | Intervention type | Outcomes measured | Key findings relevant to scoping review research question | | | | | | | |
| --- | --- | --- | --- | --- | --- | --- | --- | --- | --- | --- | --- | --- | --- |
|  |  |  |  |  |  | Administration time and Administrator | Ease of use | Completeness of data | Responsiveness / sensitivity to change | Variables (subscales) | Cut off used for agitation and other behaviours | Accuracy (sensitivity & specificity, assessed against gold standard used in study) | Reliability and validity |
