# Supplementary material for: Identifying instruments for measuring agitation and other non-cognitive symptoms in people with advanced dementia in residential settings: a scoping review protocol
Source: BMJ Open. 2025 Aug 12;15(8):e096540. doi: 10.1136/bmjopen-2024-096540 (PMC12352213; doi:10.1136/bmjopen-2024-096540)
Supplement: Supplementary file 2 [file bmjopen-2024-096540.reviewer_comments.pdf]

PEER REVIEW HISTORY

BMJ Open publishes all reviews undertaken for accepted manuscripts. Reviewers are asked to complete a checklist review form and are provided with free text boxes to elaborate on their assessment. These free text comments are reproduced below.

ARTICLE DETAILS

Title (Provisional)

Identifying instruments for measuring agitation and other non-cognitive symptoms in people with advanced dementia in residential settings: a scoping review protocol

Authors

Faherty, Mary; O Mahony, Lauren; Cornally, Nicola; Brady, Noeleen; Dalton O' Connor, Caroline; Fox , Siobhan; Hartigan, I; van den Broek, Brenda; van der Steen, Jenny Theodora; Timmons, Suzanne

---

VERSION 1 - REVIEW

**Reviewer**	**1**
**Name**	**Bergh, Sverre**
**Affiliation**	**Innlandet Hospital Trust**
**Date**	**07-Mar-2025**
**COI**	**None**

Thank you for the opportunity to review your manuscript. The research question is important, and your described methodology is sound.

I have some comments that may improve your manuscript, but overall, the quality of the proposed scoping review is good.

1. I expect that you will only include English papers. Or will you include papers in other languages? Please clarify

2. Page 2, line 54. Is it necessary with decimals in the description of cumulative incidence of agitation?

3. Page 3, line 54: What is meant with the abbreviation SR?

4. Page 6, line 41-52. Please include information that the draft carting table is found in the suppl. Material

---

VERSION 1 - AUTHOR RESPONSE

Reviewer 1:

Comments to the Author:

Thank you for the opportunity to review your manuscript. The research question is important, and your described methodology is sound. I have some comments that may improve your manuscript, but overall, the quality of the proposed scoping review is good.

1. I expect that you will only include English papers. Or will you include papers in other languages? Please clarify

We thank the reviewer for their feedback. We plan to include any non-English papers that are found through our (English) search terms. We have adjusted the inclusion criteria source characteristics to note 'articles in any language'.

2. Page 2, line 54. Is it necessary with decimals in the description of cumulative incidence of agitation?

We thank the reviewer for their feedback. We have rounded the figures to the nearest whole number.

3. Page 3, line 54: What is meant with the abbreviation SR?

We thank the reviewer for their feedback. We have deleted the acronym and replaced it with 'systematic review'.

4. Page 6, line 41-52. Please include information that the draft carting table is found in the suppl. Material

We thank the reviewer for their feedback. We have amended the text to confirm a draft charting table is included in supplementary file 1.

Reviewer: 1

If you have selected 'Yes' above, please provide details of any competing interests.: No competing interests

We thank the reviewer for their feedback. We have amended to confirm: 'No competing interests.'
